# Supplementary material for: UGT1A1 variants in Chinese Uighur and Han newborns and its correlation with neonatal hyperbilirubinemia
Source: PLoS One. 2022 Dec 15;17(12):e0279059. doi: 10.1371/journal.pone.0279059 (PMC9754166; doi:10.1371/journal.pone.0279059)
Supplement: S1 File — (PDF) [file pone.0279059.s002.pdf]

Ethic proof

To whom it may concern;

Clinical case study part of the project " The role of UGT1A1 gene promoter polymorphism and epigenetic modification in neonatal hyperbilirubinemia", full respect for patients and their family members informed choice, using retrospective analysis method in the process of study, does not affect the patient diagnosis and treatment process, the patient does not assume any additional risk, and the results are helpful for guiding clinical work.

After discussion and unanimous approval by all members of the Ethics Committee of the School of Medicine of Yangtze University, the clinical research part of the project meets the requirements of medical ethics, and the application is agreed.

Ethics Committee of the School of Medicine of Yangtze University

March, 2018

Sign of research members:

Sign of cooperated institution:

Maternity and Child Health Care Hospital of Urumqi

签字和盖章页

接收编号：8180061860

申 请 人： 杨辉 依托单位： 长江大学

项目名称： UGT1A1基因启动子多态及表观遗传学修饰在新生儿高胆红素血症发病中的作用研究

资助类别： 青年科学基金项目 亚类说明：

附注说明：

申请人承诺：

我保证申请书内容的真实性。如果获得资助，我将履行项目负责人职责，严格遵守国家自然科学基金委员会的有关规定，切实保证研究工作时间，认真开展工作，按时报送有关材料。若填报失实和违反规定，本人将承担全部责任。

签字：

项目组主要成员承诺：

我保证有关申报内容的真实性。如果获得资助，我将严格遵守国家自然科学基金委员会的有关规定，切实保证研究工作时间，加强合作、信息资源共享，认真开展工作，及时向项目负责人报送有关材料。若个人信息失实、执行项目中违反规定，本人将承担相关责任。

编号	姓名	工作单位名称（应与加盖公章一致）	证件号码	每年工作时间（月）	签字
1	郑兵	长江大学	421081198209264271	8	李慧君
2	李慧君	乌鲁木齐市妇幼保健院	654101198005260024	6	
3	习珊珊	长江大学	429005199003255228	8	
4	曾梦柳	长江大学	420982199109017248	8	
5	代文成	乌鲁木齐市妇幼保健院	652701198202252518	6	代文成
6	孙云丰	长江大学	42108719890906683X	8	
7	包建兵	长江大学	421221199210041832	8	
8					
9					

依托单位及合作研究单位承诺：

已按填报说明对申请人的资格和申请书内容进行了审核。申请项目如获资助，我单位保证对研究计划实施所需要的人力、物力和工作时间等条件给予保障，严格遵守国家自然科学基金委员会有关规定，督促项目负责人和项目组成员以及本单位项目管理部门按照国家自然科学基金委员会的规定及时报送有关材料。

依托单位公章 合作研究单位公章1 合作研究单位公章2

日期： 日期： 日期：

乌鲁木齐市妇幼保健院伦理审查批准表

伦理审批编号：XJFYLL2017002

申报项目名称	汉维族新生儿黄疸分子流行病学调查研究		
申报科室	产前诊断中心	项目所属专业	产前诊断
科室负责人/职称	李慧君/主治医师	联系方式	15609912028
项目负责人/职称	代文成/主管技师	联系方式	15999162993
需要涉及的伦理问题	患者知情同意		
提供审查资料	项目申请书		
主要参加单位	乌鲁木齐市妇幼保健院		

伦理委员会审查意见

经本伦理委员会审查，同意☑ 不同意□ 进行该项临床实践/研究。

意见及建议：无☑ 有□

伦理委员会主任签字：[illegible]

伦理委员会盖章：

日期：2017.5.16

备注：（请仔细阅读）

1. 本批件将在伦理委员会备案。
2. 已批准项目需遵循本伦理委员会批准的方案执行。
3. 伦理委员会有权对研究过程进行跟踪评价。
4. 项目内容变更，暂停或提前终止临床研究，请及时报告伦理委员会。
5、发生严重不良事件及影响研究风险收益比的非预期事件，请及时报告伦理委员会。
6、完成临床研究，请提交结题报告供伦理委员会审查。
