# Supplementary material for: UGT1A1 variants in Chinese Uighur and Han newborns and its correlation with neonatal hyperbilirubinemia
Source: PLoS One. 2022 Dec 15;17(12):e0279059. doi: 10.1371/journal.pone.0279059 (PMC9754166; doi:10.1371/journal.pone.0279059)
Supplement: Attachment — Submitted filename: response to reviewers2-ok.docx [file pone.0279059.s005.docx]

**Response to reviewers**

**Additional Editor Comments:**

Please outline how ethnic group was determined in your Methods section. Since any method of identifying an ethnic group suffers from limitations, please discuss the limitations of the method(s) used for this manuscript in your Discussion section.

**Answer**：Thank you for your suggestion. We have clarified this question in the revised manuscript. In this study, blood samples (umbilical cord and peripheral blood species) of healthy unrelated newborns from the two main ethnic groups (Uygur and HAN) were collected. The ethnic composition of the newborns was determined according to the ethnic identity of his father and mother. Both their parents were Uygur or Han were included in this study. In fact, intermarriage between Uyghur and Han is rare, though it is allowed by the law of China.

**Reviewer #1**: Only remaining comment is on Table 3. It's unclear why some rows have p-values but not odds ratios (and vice versa). Also, the inclusion of 0.00 odds ratios is confusing, is this meant to indicate there were equal numbers of cases/controls with that particular genotype? It might be clearer to list the counts, and then the p-value next to the Odds ratio. If I'm misunderstanding, perhaps horizontal lines would help clarify how the data is meant to align.

**Answer:** Thank you for your question. We have adjusted table 3 and make it easier to see as suggested.
