# Supplementary material for: UGT1A1 variants in Chinese Uighur and Han newborns and its correlation with neonatal hyperbilirubinemia
Source: PLoS One. 2022 Dec 15;17(12):e0279059. doi: 10.1371/journal.pone.0279059 (PMC9754166; doi:10.1371/journal.pone.0279059)
Supplement: Attachment — Submitted filename: response to reviewers-ok.docx [file pone.0279059.s006.docx]

**Response to the academic editor and** **reviewers**

1. The concerns previously noted by the reviewer have been addressed. However, my previous concerns about how ethnicity was determined have not been addressed satisfactorily. It is indicated that "the ethnic composition of the newborns was determined according to the ethnic identity of his father and mother", but how the ethnic composition was determined remains unclear. Please update your Methods section to provide enough details of how ethnicity was determined, such that another researcher could repeat the procedures with the information provided. In your Discussion section, please discuss whether it was possible that the ethnicity of any of the participants was incorrect

**Answer**：Thank you for your suggestion. We have clarified this question more precisely in the revised manuscript. The ethnic of the newborns was inferred from the demographic information (name and race) of their parents by reviewing the clinical record. Both their parents were Uygur or Han were included in this study. In fact, there are so many differences between Uygur and Han, such as their appearance, costume, linguistic, naming way and so on, so that it was not difficult to differential one from the other. Moreover, the demographic information of the study participants was carefully check not less than three times by us, it was not possible that mistaken the ethnicity of any of the participants.

**Journal Requirements:**

Please review your reference list to ensure that it is complete and correct. If you have cited papers that have been retracted, please include the rationale for doing so in the manuscript text, or remove these references and replace them with relevant current references. Any changes to the reference list should be mentioned in the rebuttal letter that accompanies your revised manuscript. If you need to cite a retracted article, indicate the article’s retracted status in the References list and also include a citation and full reference for the retraction notice.

**Answer**：No change.
