# Supplementary material for: UGT1A1 variants in Chinese Uighur and Han newborns and its correlation with neonatal hyperbilirubinemia
Source: PLoS One. 2022 Dec 15;17(12):e0279059. doi: 10.1371/journal.pone.0279059 (PMC9754166; doi:10.1371/journal.pone.0279059)
Supplement: Attachment — Submitted filename: response to reviewers-final.docx [file pone.0279059.s007.docx]

**Response to the editor**

1) It is noted that "The ethnic of the newborns was inferred from the demographic information (name and race) of their parents by reviewing the clinical record.". Please indicate how race/ethnicity was originally determined when clinical data was collected - please indicate whether ethnicity was self-reported by patients, or whether this was inferred by clinicians.

**Answer**：Thank you for your suggestion. Demographic information including the race/ ethnicity was self-reported by the patients on their admission. We have detail this in the revised manuscript and marked red.

2) Suggest to revise the sentence "Both their parents were Uygur or Han were included in this study." to " If both their parents were Uygur or Han newborns were included in this study."

**Answer**：Thank you for your suggestion. I have done the modification as you suggested in the revised manuscript.

3) Please remove this sentence "In fact, there are so many differences between Uygur and Han, such as their appearance, costume, linguistic, naming way and so on, so that it was not difficult to differential one from the other"

**Answer**：Thank you for your suggestion. We have done the revision as suggested.

4) Please ensure the spelling of Uyghur is consistent throughout the manuscript, as several spellings are currently used (e.g. Uighur in the title and Uygur in the abstract and other sections of the manuscript)

**Answer**：Thank you for your suggestion. I have done the modification as suggested in the revised manuscript.
